# Intrinsic capacity and survival among older adults in India: LASI-DAD study (wave 1 and wave 2)

**DOI:** 10.1016/j.lansea.2026.100775

**Published:** 2026-05-09

**Authors:** Abhijith Rajaram Rao, Manjusha Bhagwasia, Akshata Rao, Mujtaba Waris, Sakthi Kiruthika, Sudeep George, Mihir Vedula, Shreya Biswal, Richa Mallick, Tejas Shivamogga Ranganatha, Avinash Chakrawarty, Jinkook Lee, Sharmista Dey, Aparajit Ballav Dey

**Affiliations:** a Department of Geriatric Medicine, National Centre for Ageing, All India Institute of Medical Sciences, New Delhi, India; b Department of Paediatrics, All India Institute of Medical Sciences, New Delhi, India; c NHS Trust, University Hospitals Sussex, Brighton, United Kingdom; d Department of Economics, University of Southern California, USA; e Department of Biophysics, All India Institute of Medical Sciences, New Delhi, India; f Venu Geriatric Care Center, New Delhi, India

**Keywords:** Intrinsic capacity, Healthy ageing, Mortality, Cognition, Nutrition, Locomotion, Indian older adults

## Abstract

**Background:**

With increasing longevity, the focus of healthcare for older adults is shifting from a disease-centric model to a more holistic view of functional capacity. This study investigates the relationship between intrinsic capacity (IC)—a measure of combined physical and mental abilities—and survival among older adults in India.

**Methods:**

We analysed data from 4096 community-dwelling individuals aged 60 and older who participated in the Longitudinal Aging Study in India–Diagnostic Assessment of Dementia (LASI-DAD). IC was evaluated across six domains: cognition, mood, nutrition, locomotion, vision, and hearing. An overall IC score was created by aggregating the z-scores from these domains. Cox Model was used to determine if IC was a predictor of mortality, adjusting for confounding factors.

**Findings:**

During the follow-up period, 951 participants (23.2%) died. A higher IC score was associated with a lower risk of death (Hazard Ratio (HR): 0.89; 95% CI: 0.86–0.91). The risk of mortality increased progressively with the number of impaired intrinsic capacity domains: participants with one impaired domain had a HR of 1.48 (95%CI 1.26–1.75); two impaired domains, HR: 2.10 (95%CI 1.67–2.65); three impaired domains, HR: 1.71 (95%CI 1.14–2.57); and four impaired domains, HR: 3.15 (95%CI 1.53–6.46). Of the six domains, cognition, nutrition, and locomotion were identified as the strongest predictors of survival. Vision impairment did not show a independent association with mortality.

**Interpretation:**

Older adults in India with higher overall intrinsic capacity—particularly better cognition, good nutrition, and better locomotor capacity—had a lower risk of death. These findings highlight the importance of looking at functional abilities, not just diseases, when planning health care and interventions for ageing populations.

**Funding:**

LASI-DAD is funded by the 10.13039/100000049National Institute on Aging, the 10.13039/100000002National Institutes of Health (R01 AG051125, U01AG064948).

**Research in context**

**Evidence before this study**

Most evidence on intrinsic capacity (IC) and mortality comes from high-income countries, such as the English Longitudinal Study of Ageing (ELSA) and the Health and Retirement Study (HRS), showing that higher IC is linked to lower risk of death. A few studies from low- and middle-income countries, including India, have measured IC, but none have examined its relationship with mortality over time. Previous work has largely focused on diseases rather than overall functional ability.

**Added value of this study**

With data from a sample of older adults in India, this study looks at IC across six domains—cognition, mood, nutrition, locomotion, vision, and hearing—and shows how both overall IC and specific domain deficits predict survival. This provides longitudinal evidence from India validating the WHO IC framework in a low- and middle-income context.

**Implications of all the available evidence**

These findings support focusing on function, rather than just diseases, in older adults. Measuring IC could help identify people at higher risk and guide early interventions to maintain or improve functioning. This approach has potential value for policy, clinical practice, and healthy ageing strategies, especially in settings with limited healthcare resources.

## Introduction

The world’s population is ageing at an unprecedented rate, with the number of people aged 60 years and older projected to double from approximately 1 billion in 2020 to 2.1 billion by 2050^1^ This demographic shift presents significant challenges for health systems, particularly in low- and middle-income countries (LMICs), where resources for older adults are often limited. In response, the WHO has proposed the *Healthy Ageing* framework, which shifts the focus from disease-centred care to the preservation of functional ability—the capacity of individuals to do and be what they value. Central to this framework is intrinsic capacity (IC), defined as the composite of an individual’s physical and mental capacities.^2^ By enabling fine-grained assessment and regular monitoring, IC offers the potential to detect early declines before clinical symptoms appear, creating opportunities for timely preventive interventions that could delay or avert geriatric syndromes and reduce future healthcare demands.^3^^,^^4^

Evidence from longitudinal studies in high-income countries (HICs) suggests that older adults with higher intrinsic capacity are more likely to experience favourable health trajectories, including a lower risk of functional decline, falls, institutionalisation, and death.^5^, ^6^, ^7^, ^8^ Importantly, declines in IC often precede dependency and can more reliably predict adverse outcomes than chronological age alone.^9^

India has one of the fastest-growing older populations in the world, with more than 300 million people expected to be aged 60 years or older by 2050.^10^ Despite this demographic shift, little is known about how IC influences survival in the Indian context. While a few cross-sectional studies exist, these are limited to hospital-based samples^11^ or specific communities.^12^ Nationally representative research examining the link between IC and survival is scarce.^13^ Most existing evidence is derived from HICs and may not be generalisable to India, where the social, nutritional, and healthcare environments are markedly different.^14^ Although individual elements of intrinsic capacity—such as impaired locomotion, undernutrition, and cognitive decline—have been linked to higher mortality in some Indian studies, no large-scale, nationally representative research has examined the association between overall IC, its constituent domains, and survival outcomes.

The Longitudinal Aging Study in India—Diagnostic Assessment of Dementia (LASI-DAD) provides a unique opportunity to fill this evidence gap.^15^ This large, nationally representative survey of older adults collects comprehensive information across all domains of intrinsic capacity, along with prospective mortality data. While associations between IC and survival have been demonstrated predominantly in high-income setting, evidence from low- and middle-income countries remains limited. Given the distinct life-course exposures, socioeconomic gradients, disease burden, and health-system context in India, contextual validation of the IC framework is essential for informing healthy ageing policy and clinical practice in such settings.

In this study, we aimed to (1) examine the association between baseline IC and all-cause mortality in older Indian adults, and (2) identify which specific IC domains, as quantified by z-scores, are most strongly associated with survival. We hypothesised that individuals with higher baseline IC would have a lower risk of death, independent of demographic characteristics and comorbidities.

## Methods

The Longitudinal Aging Study in India (LASI) is a nationally representative survey of 72,250 community-dwelling individuals aged ≥ 45 years. The LASI–Diagnostic Assessment of Dementia (LASI-DAD) is a sub-sample of LASI designed to study late-life cognition and dementia in India. For the present analysis, we included all LASI-DAD participants aged ≥ 60 years at baseline.

Although the main LASI survey includes a large nationally representative cohort, only one wave of LASI has been fully completed to date, which precludes longitudinal survival analyses. In contrast, LASI-DAD provides both baseline (Wave 1) and completed follow-up (Wave 2) data, along with confirmed mortality ascertainment. Therefore, LASI-DAD was chosen as it uniquely enabled longitudinal evaluation of intrinsic capacity and mortality within the Indian ageing population.

LASI-DAD Wave 1 was conducted between 2017 and 2019 and enrolled 4096 older adults. Wave 2 was conducted between 2022 and 2024 and was designed to follow up all Wave 1 respondents, with no additional inclusion or exclusion criteria beyond baseline eligibility, as described previously. In this study, intrinsic capacity and covariates were assessed at Wave 1, and all-cause mortality was ascertained during Wave 2 follow-up. The sampling strategy and study design have been described in detail elsewhere.^16^^,^^17^

Intrinsic capacity was assessed across the following domains: cognition, mood, nutrition, locomotion, vision, and hearing.^18^ Cognition was evaluated at baseline using a standardised total cognition score derived from a battery of neuropsychological tests covering multiple domains (Supplementary Table S1). These included measures of orientation, immediate and delayed word recall, story recall (immediate and delayed), verbal fluency, language comprehension, object naming, visuospatial ability, and executive function. Each individual test score was first standardised, and the total cognition score was generated by summing the standardised components. Z-scores were calculated using the study baseline population as the reference distribution, and higher score indicated better cognitive performance.

Mood was assessed using the Center for Epidemiologic Studies Depression Scale (CES-D).^19^ As higher CES-D scores indicate worse mood, the z-score was multiplied by −1 to ensure that higher values reflected better emotional well-being (Supplementary Table S2). Nutrition was measured using the Mini Nutritional Assessment (MNA) full form.^20^ The total MNA score was transformed into a z-score, with higher values reflecting better nutritional health. Locomotor function was assessed using gait speed over a 4-m walk. Participants were instructed to walk at their usual pace, and time was recorded using a stopwatch. Two trials were performed, and the average time was used to calculate gait speed (m/s). This value was then standardised to a z-score.

Vision was assessed separately for near and distance acuity in both eyes using standard charts. All measurements were converted to the logarithm of the minimum angle of resolution (logMAR), which provides a continuous, linear scale of visual function. Because higher logMAR scores indicate poorer vision, values were multiplied by −1 so that higher numbers indicated better vision. The four adjusted logMAR values (right and left eye for both near and distance) were summed to create a composite vision score, which was then standardised as a z-score.

Hearing was evaluated in each ear using the HearCheck Screener, which presents six tones at fixed frequencies and intensities. One point was awarded for each tone correctly detected, and scores from both ears were summed. The combined hearing score was transformed into a z-score, with higher values representing better hearing ability. CES-D, MNA, gait speed, vision test and hearing screening are geriatric assessment tools which have been validated in Indian older adults.

Intrinsic capacity was operationalized in two complementary ways to capture both the overall level of functioning and the pattern of deficits across domains.

First, we created a continuous IC score. For each of the six domains, raw scores were converted into z-scores, which express how far an individual’s score lies above or below the average performance of the study population. Before combining them, all scores were aligned so that higher values always indicated better function. The six standardized domain scores were then added together to obtain a single composite IC score. Using standardized scores allows us to combine measures that are originally expressed in very different units (for example, gait speed in meters/second versus depression score counts) in a fair and comparable manner. Each domain was given equal weight in the composite score, because there is currently no validated evidence to justify prioritizing one IC domain over another.

Second, we also created a categorical measure of IC impairment. For each domain, participants were classified as either “impaired” or “not impaired” based on their z-score. A cut-off of −1.5 standard deviations below the mean was used to define impairment. This threshold represents a meaningful deviation from typical performance in the population and has been applied in previous literature. We selected a uniform z-score threshold rather than instrument-specific clinical cut-offs to ensure conceptual consistency and comparability across domains that are measured on different scales. The number of impaired domains per participant (range 0–6) was then calculated to generate the domain impairment count measure. By using both a continuous composite score and an impairment count, we were able to capture both the overall level of intrinsic capacity and the extent of domain-specific functional loss, providing a more comprehensive understanding of IC.

Participants were not excluded solely due to missing information in one or more IC domains. Domain-specific analyses were conducted using all participants with available data for that respective domain. For the continuous composite IC score, the score was calculated using all available standardized domain values for each participant.

Mortality between Wave 1 and Wave 2 was ascertained through end-of-life interviews conducted with a proxy informant, typically a close family member of the deceased participant. Interviewers recorded the month and year of death. The time-to-event variable was calculated by taking the date of the Wave 1 interview as the starting point and the reported date of death as the endpoint. Both dates were standardised to the first day of the reported month to allow uniform computation.

In participants where death occurred in the same month as the Wave 1 interview, we assigned a survival time of 0.5 months to reflect partial-month survival. When the month of death was missing but the year was reported, the death was assumed to have occurred midway through that year. For participants with missing year of death but confirmed deceased status in Wave 2, we assumed the event occurred halfway between the Wave 1 interview and the Wave 2 follow-up interview date. Of the 951 deaths observed, assumptions regarding the timing of death were required for 60 participants (6.3% of deaths; 1.5% of the total cohort), including 7 deaths occurring in the same month as the Wave 1 interview, 28 with missing year or year-and-month of death, 9 with internally inconsistent reports (death reported before the Wave 1 interview), and 16 with missing month of death (Supplementary Fig. S1). Median follow-up time was 51.94 months (IQR 50.03 to 59.99), estimated using the reverse Kaplan–Meier method.

Participants who were alive at the time of Wave 2 or for whom no death was reported but who did not complete the Wave 2 interview were treated as right-censored (Supplementary Fig. S1). In the absence of individual-level information distinguishing refusal from non-traceability, these participants were administratively censored at the end of the Wave 2 follow-up period. In a sensitivity analysis, participants without a confirmed death were censored at the midpoint between the Wave 1 interview date and the start of the Wave 2 follow-up period.

Sociodemographic characteristics included age at Wave 1, sex, education level (less than upper secondary, upper secondary or vocational, tertiary), body mass index (BMI), monthly per capita expenditure (MPCE), marital status, and place of residence (urban or rural). Medical history included self-reported diagnoses of hypertension, diabetes, cancer, chronic lung disease, stroke, neurological or psychiatric illness, and high cholesterol. Functional status was assessed using basic and instrumental activities of daily living.

### Statistical analysis

Baseline characteristics of the study population were summarised using descriptive statistics. Categorical variables were summarised as frequencies and percentages, while continuous variables were described as mean (standard deviation) or median (interquartile range), depending on their distribution. Baseline characteristics were compared between survivors and non-survivors using the Chi-square test for categorical variables and the Wilcoxon rank-sum test for continuous variables.

Time-to-event analyses were performed to examine the association between intrinsic capacity and all-cause mortality. Kaplan–Meier survival curves were constructed to visualise survival according to the number of impaired intrinsic capacity domains, and differences between groups were assessed using the log-rank test.

Cox proportional hazards regression was used to estimate hazard ratios (HR) and 95% confidence intervals (CI) for mortality. Three sequentially adjusted Cox proportional hazards models were fitted: Model 1 included the IC measure only (unadjusted). Model 2 was additionally adjusted for age and sex. Model 3 was adjusted for age, sex, BMI, hypertension, diabetes, cancer, chronic lung disease, chronic heart disease, stroke, neurological or psychiatric illness, and high cholesterol. These covariates were selected a priori based on existing literature and their availability in the LASI-DAD dataset.

These models were applied separately to four representations of intrinsic capacity:

(1) the summated IC score (continuous),
(2) the IC category representing the number of impaired domains,
(3) individual IC domain z-scores, and
(4) individual IC domains categorised as impaired or unimpaired.

To examine the independent contribution of each IC domain, an additional multivariable Cox model was fitted in which all IC domain z-scores were entered and adjusted for age, sex, BMI, hypertension, diabetes, cancer, chronic lung disease, chronic heart disease, stroke, neurological or psychiatric illness, and high cholesterol, was used to identify domains independently associated with survival. The proportional hazards assumption was assessed using Schoenfeld residual–based tests, and scaled Schoenfeld residual plots were examined for the fully adjusted model.

Sensitivity analyses were conducted to assess the robustness of the findings to censoring assumptions. Participants without confirmed death were alternatively censored at the midpoint between the Wave 1 interview date and the end of the Wave 2 follow-up period. Statistical significance was defined as a two-sided p value < 0.05. Analyses were conducted using Stata version 17 (StataCorp. 2021. Stata: Release 17. Statistical Software. College Station, TX: StataCorp LLC.), and figures were generated with GraphPad Prism version 8 (GraphPad Software, San Diego, CA, USA).

### Ethics statement

Written informed consent or thumbprint consent was obtained from all participants. For participants who had cognitive impairment, consent was obtained from a legally authorised representative. Ethical approval was granted by the Indian Council of Medical Research (ICMR) (Ref: 2202-16741/F1) and all collaborating institutions. This study was conducted and reported in accordance with the STROBE guidelines for cohort studies.

### Role of the funding source

The funders had no role in study design, data collection, data analysis, interpretation, writing of the report.

## Results

We analysed data from 4096 participants, with a median (IQR) age at baseline of 68 (64–74) years, and 2206 (53.86%) were female. Among them, 3145 (76.8%) were alive or censored at the end of follow-up, and 951 (23.2%) had died. Bivariate analysis showed that the median age was higher among non-survivors compared to survivors (72 years [IQR: 67–80] versus 67 years [IQR: 63–72], p < 0.001). Mortality was higher among males than females (27.6% versus 19.5%, p < 0.001). Educational attainment showed an inverse relationship with mortality, with the highest proportion of deaths among those with less than upper secondary education (p = 0.001). Non-survivors had a lower median BMI (20.78 [IQR: 17.76–20.78]) than survivors (22.39 [IQR: 19.05–25.89], p < 0.001). Marital status was also associated with survival (p < 0.001), with widowed participants exhibiting a higher mortality rate (27.9%) (Table 1).

**Table 1:** Baseline characteristics of participants between groups (n = 4096).

Variable	Survivors (n = 3145)	Non-survivors (n = 951)	p-value
Age [median (IQR)]	67 (63–72)	72 (67–80)	<0.001
Sex			
Male	1369 (72.43%)	521 (27.57%)	<0.001
Female	1776 (80.51%)	430 (19.49%)	
Education			
Less than upper secondary	2327 (75.55%)	753 (24.45%)	0.001
Upper secondary and vocational training	663 (79.50%)	171 (20.50%)	
Tertiary	155 (85.16%)	27 (14.84%)	
Body mass index	22.39 (19.05–25.89)	20.78 (17.76–20.78)	<0.001
MPCE			
Poorest	600 (76.53%)	184 (23.47%)	0.265
Poorer	633 (76.82%)	191 (23.18%)	
Middle	663 (76.47%)	204 (23.53%)	
Richer	606 (74.91%)	203 (25.09%)	
Richest	636 (79.60%)	163 (20.40%)	
Marital status			
Married	2103 (79.60%)	539 (20.40%)	<0.001
Partnered	21 (70.00%)	9 (30.00%)	
Separated	7 (50.00%)	7 (50.00%)	
Divorced	11 (57.89%)	8 (42.11%)	
Widowed	974 (72.15%)	376 (27.85%)	
Never married	29 (70.73%)	12 (29.27%)	
Place of residence			
Urban	1185 (75.91%)	376 (24.09%)	0.301
Rural	1960 (77.32%)	575 (22.68%)	
Comorbidities			
Hypertension	1205 (75.17%)	398 (24.83%)	0.032
Diabetes	503 (70.15%)	214 (29.85%)	<0.001
Cancer	23 (60.53%)	15 (39.47%)	0.016
Chronic lung disease	242 (67.60%)	116 (32.40%)	<0.001
Chronic heart disease	199 (71.84%)	78 (28.16%)	0.037
Stroke	68 (56.67%)	52 (43.33%)	<0.001
Neurological and psychiatric illness	92 (66.19%)	47 (33.81%)	0.002
High cholesterol	153 (80.53%)	37 (19.47%)	0.228
Functional status			
ADL	6 (4–6)	5 (2–6)	<0.001
IADL	6 (4–7)	4 (1–6)	<0.001
Intrinsic capacity and domains			
Summated IC score	0.55 (−1.68 to 2.71)	−1.62 (−3.95 to 0.64)	<0.001
Cognition z score	0.15 (−0.51 to 0.78)	−0.36 (−1.14 to 0.37)	<0.001
Mood z score	0.18 (−0.55 to 0.74)	−0.01 (−0.74 to 0.55)	<0.001
Nutrition z score	0.20 (−0.51 to 0.77)	−0.22 (−1.08 to 0.34)	<0.001
Locomotor z score	0.07 (−0.52 to 0.75)	−0.47 (−1.06 to 0.29)	<0.001
Vision z score	0.18 (−0.57 to 0.71)	−0.06 (−0.84 to 0.70)	<0.001
Hearing z score	0.17 (−0.59 to 0.56)	−0.21 (−0.98 to 0.17)	<0.01

Among comorbidities, hypertension (p = 0.032), diabetes (p < 0.001), cancer (p = 0.016), chronic lung disease (p < 0.001), chronic heart disease (p = 0.037), stroke (p < 0.001), and neurological or psychiatric illness (p = 0.002) were more prevalent among non-survivors. High cholesterol was not associated with mortality (p = 0.228). Non-survivors had lower median ADL (5 versus 6) and IADL (4 versus 6) scores (both p < 0.001).

In univariable Cox proportional hazards analyses of baseline demographic and clinical variables, age, sex, hypertension, diabetes, cancer, chronic lung disease, stroke and neurological and psychiatric illness were associated with mortality (Supplementary Table S3).

Domain-specific data were available for cognition (n = 4095), mood (n = 4096), nutrition (n = 3623), locomotor capacity (n = 3619), vision (n = 3716), and hearing (n = 4096). For analyses using the composite IC score, the score was calculated using available standardized domain values for each participant, such that missing data in a single domain did not automatically exclude individuals from analysis. Median follow-up time was 51.94 months (IQR 50.03 to 59.99).

In unadjusted Cox models (Model 1), higher summated IC scores were strongly associated with lower mortality risk (HR: 0.85; 95% CI: 0.83–0.86). This association remained statistically significant after adjustment for age and sex (Model 2: HR: 0.87; 95% CI: 0.85–0.89) and further adjustment for BMI and comorbidities (Model 3: HR: 0.89; 95% CI: 0.86–0.91) (Table 2).

**Table 2:** Survival analysis of summated IC score and individual domain.

Variable	Model 1^a^		Model 2^b^		Model 3^c^	
	HR (95% CI)	p-value	HR (95% CI)	p-value	HR (95% CI)	p-value
Intrinsic capacity	0.85 (0.83–0.86)	<0.001	0.87 (0.85–0.89)	<0.001	0.89 (0.86–0.91)	<0.001
Cognition	0.61 (0.57–0.65)	<0.001	0.69 (0.65–0.73)	<0.001	0.70 (0.65–0.76)	<0.001
Mood	0.84 (0.78–0.89)	<0.001	0.85 (0.79–0.90)	<0.001	0.86 (0.80–0.92)	<0.001
Nutrition	0.67 (0.63–0.72)	<0.001	0.71 (0.66–0.77)	<0.001	0.74 (0.68–0.80)	<0.001
Locomotor	0.64 (0.59–0.69)	<0.001	0.72 (0.66–0.78)	<0.001	0.73 (0.67–0.79)	<0.001
Vision	0.86 (0.80–0.92)	<0.001	0.93 (0.87–0.99)	0.029	0.94 (0.88–1.01)	0.099
Hearing	0.69 (0.65–0.74)	<0.001	0.79 (0.75–0.85)	<0.001	0.83 (0.77–0.89)	<0.001

a Unadjusted.

b Adjusted for age and sex.

c Adjusted for age, sex, BMI, hypertension, diabetes, cancer, chronic lung disease, chronic heart disease, stroke, neurological and psychiatric illness, high cholesterol.

**Fig. 1:**
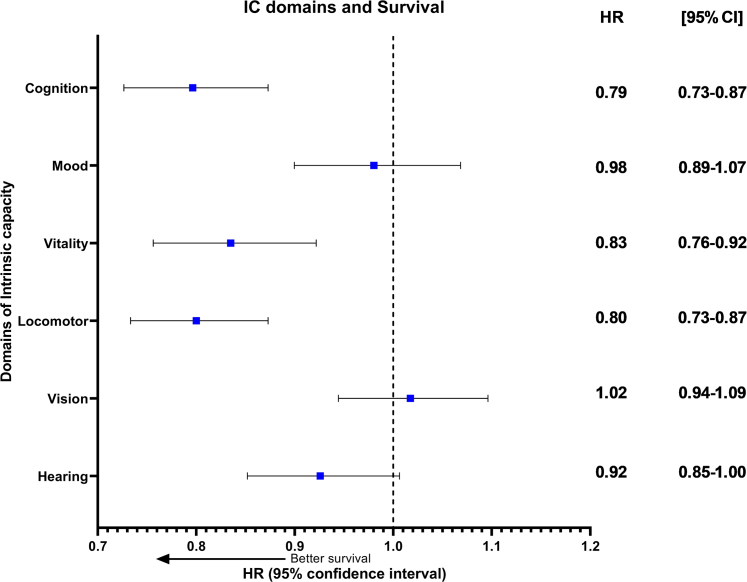
Forrest plot showing results of survival analysis including all domains of IC (adjusted for each other and age, sex, BMI, hypertension, diabetes, cancer, chronic lung disease, chronic heart disease, neurological and psychiatric illness, high cholesterol).

All IC domains, apart from vision, showed statistically significant inverse associations with mortality after full adjustment. The strongest associations were observed for cognition (HR: 0.70; 95% CI: 0.65–0.76), nutrition (HR: 0.74; 95% CI: 0.68–0.80), and locomotor function (HR: 0.73; 95% CI: 0.67–0.79). When all domains were included in the model (Fig. 1), we found that cognition (HR: 0.79; 95% CI: 0.72–0.87), nutrition (HR: 0.83; 95% CI: 0.76–0.92), and locomotor function (HR: 0.80; 95% CI: 0.73–0.87) were associated with survival.

**Fig. 2:**
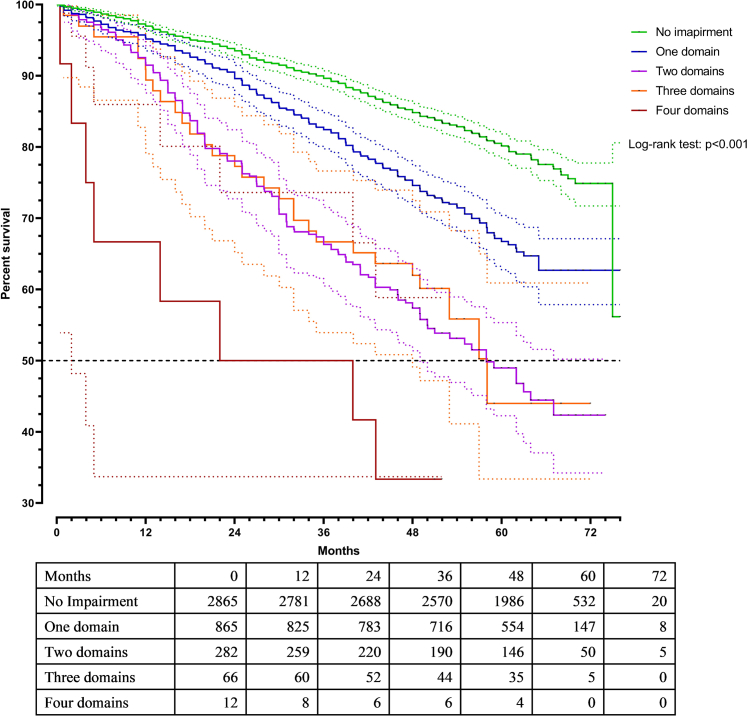
Kaplan–Meier estimates of survival by number of impaired IC domains.

Kaplan–Meier curves demonstrated progressively lower survival with increasing numbers of impaired IC domains, and survival distributions differed between group according to the log-rank (Mantel–Cox) test (log-rank χ^2^= 242.2 p-value < 0.001) (Fig. 2). When IC was classified by the number of impaired domains, a dose–response relationship existed between impairment burden and mortality risk (p < 0.001). Compared to participants with no impairment, those with one impaired domain had a 48% higher adjusted risk of death (HR: 1.48; 95% CI: 1.26–1.75), while those with four impaired domains faced more than a threefold increase in risk (HR: 3.15; 95% CI: 1.53–6.46) (Table 3). Median survival decreased sharply with increasing impairment, from “not reached” for those with ≤1 impaired domain to 22.0 months for those with four impairments.

**Table 3:** Survival analysis of intrinsic capacity and domains defined as categorical variable.

Variable	Median survival (months)	Model 1^a^		Model 2^b^		Model 3^c^	
		HR (95% CI)	p-value	HR (95% CI)	p-value	HR (95% CI)	p-value
Intrinsic capacity							
No impairment	Not reached	1 (ref)		1 (ref)			
One impaired	Not reached	1.76 (1.52–2.05)	<0.001	1.55 (1.33–1.81)	<0.001	1.48 (1.26–1.75)	<0.001
Two impaired	57.95	3.36 (2.78–4.06)	<0.001	2.47 (2.02–3.01)	<0.001	2.10 (1.67–2.65)	<0.001
Three impaired	58.05	3.12 (2.14–4.53)	<0.001	1.74 (1.18–2.56)	0.005	1.71 (1.14–2.57)	0.009
Four impaired	22.01	7.26 (3.61–14.60)	<0.001	3.91 (1.93–7.94)	<0.001	3.15 (1.53–6.46)	0.002
Individual domains	N						
Cognition	N = 4095	3.35 (2.82–3.98)	<0.001	2.25 (1.87–2.71)	<0.001	2.09 (1.69–2.59)	<0.001
Mood	N = 4094	1.51 (1.22–1.87)	<0.001	1.49 (1.19–1.85)	<0.001	1.49 (1.17–1.89)	0.001
Nutrition	N = 3623	2.22 (1.82–2.71)	<0.001	1.93 (1.58–2.36)	<0.001	1.68 (1.37–2.07)	<0.001
Locomotor	N = 3619	2.49 (2.01–3.09)	<0.001	1.48 (1.17–1.87)	0.001	1.33 (1.04–1.70)	0.022
Vision	N = 3716	1.55 (1.24–1.93)	<0.001	1.22 (0.97–1.51)	0.080	1.14 (0.91–1.44)	0.249
Hearing	N = 4096	2.05 (1.69–2.49)	<0.001	1.54 (1.26–1.87)	<0.001	1.31 (1.03–1.66)	0.028

a Unadjusted.

b Adjusted for age and sex.

c Adjusted for age, sex, BMI, hypertension, diabetes, cancer, chronic lung disease, chronic heart disease, stroke, neurological and psychiatric illness, high cholesterol.

For individual domains, impairment in cognition, mood, nutrition, locomotor function, and hearing were linked to increased mortality risk after full adjustment, with the highest hazard observed for impaired cognition (HR: 2.09; 95% CI: 1.69–2.59). Vision impairment showed no statistically significant association after adjustment (p = 0.249).

In supplementary analyses, tests based on Schoenfeld residuals indicated no violation of the proportional hazards assumption across the Cox models for total intrinsic capacity and individual domains, as shown in Supplementary Tables S4 and S5 Deviance residuals versus linear predictor plots and scaled Schoenfeld residual plots further supported model adequacy (Supplementary Figs. S2–S6).

We conducted sex-stratified Cox proportional hazards analyses for both the continuous summated IC score and the individual domains, as well as for the categorical IC impairment measure; proportional hazards tests are also reported for these models (Supplementary Tables S6–S8). Overall, the associations were broadly consistent across sexes, with higher intrinsic capacity associated with lower mortality in both men and women. Domain-specific categorical analyses were also performed (Supplementary Tables S9–S11). Cognition, locomotor function, and nutrition contributed most strongly to mortality risk. These findings suggest that the relationship between intrinsic capacity and survival is generally consistent across sexes, although some domain-specific effect sizes differed slightly between men and women.

Sensitivity analyses using alternative censoring assumptions showed findings consistent with the primary analyses, with similar effect sizes for the summated intrinsic capacity score and individual domains (Supplementary Tables S12 and S13). Proportional hazards tests based on Schoenfeld residuals indicated no evidence of violation of model assumptions (Supplementary Tables S14 and S15).

## Discussion

In this cohort of older Indian adults, we found that higher IC, measured as a total score across six domains, was strongly associated with lower mortality risk over a median follow-up of about five years. This association persisted after adjusting for demographic factors, body mass index, and multiple chronic conditions. Consistent with findings from longitudinal studies conducted largely in high-income settings, cognition, nutrition, and locomotor function stood out as the strongest independent predictors of survival, both as continuous and categorical measures. Additionally, we observed a clear dose–response relationship between the number of impaired domains and mortality risk, emphasising the cumulative effect of functional decline on survival. Importantly, these findings provide representative evidence from India, supporting the applicability and external validity of the World Health Organisation’s framework of IC in a low-and middle-income country context, and highlight its potential utility for community-based risk stratification and preventive geriatric care.

Community-dwelling older adults with a higher overall IC score at baseline had a lower risk of death in the final model (HR: 0.89, p < 0.001). Our findings support and extend a growing body of evidence indicating that higher IC predicts improved survival in later life. Recent longitudinal studies, including a Chinese cohort showing that declines in IC predict 8-year mortality and a multi-study systematic review and meta-analysis, reinforce the inverse relationship between IC and mortality observed in our analysis.^21^^,^^22^ In contrast to our study, the Beijing Longitudinal Study of Ageing included an older cohort (mean age 73.8 years versus 68 years in our study) and had a longer follow-up duration of eight years.^21^ Furthermore, the operationalisation of IC differed: the Beijing study defined IC across five domains, each with a score from 0 to 1, with higher scores reflecting better capacity. Their Cox proportional hazards models was adjusted only for age and sex, whereas our models additionally adjusted for comorbidities and BMI, which may partly explain differences in effect estimates.

The meta-analysis by Sánchez-Sánchez et al.,^22^ which included 37 studies (n = 2,06,693), highlighted substantial heterogeneity in IC measurement and its association with outcomes. Only three studies were eligible for pooled meta-analysis examining IC trajectories and mortality risk, demonstrating significantly higher survival among individuals with higher IC (HR 0.57, 95% CI 0.51–0.63). Across included studies, 17 operationalised IC as the sum of impaired domains (a discrete variable), whereas 16 used a composite continuous score, and 34 studies assessed IC across at least four domains. Considerable variability was also observed in the assessment tools used. Locomotion was most commonly evaluated using gait speed (15 studies) or the five-times sit-to-stand test (13 studies); cognition was assessed using the Mini-Mental State Examination (MMSE) or Montreal Cognitive Assessment (29 studies) or memory-based measures (6 studies). The psychological domain was typically measured using the Geriatric Depression Scale (16 studies) or the CES-D (14 studies). Sensory function was primarily assessed through self-reported visual or hearing limitations (26 studies), while the Mini Nutritional Assessment (MNA) was the most frequently used tool for evaluating the nutrition domain (14 studies).

Evidence from Singapore’s Longitudinal Ageing Study used 12 domain-specific measures to construct multiple composite IC indices and confirmed the validity of the multi-domain IC index, demonstrating that lower IC was consistently associated with adverse outcomes, including mortality.^23^ In this cohort of 2906 adults aged ≥ 55 years, measures such as Timed Up-and-Go, logMAR vision, and MMSE showed the strongest contributions to the overall IC construct, and the predictive performance for mortality improved as additional domains were incorporated (area under the curve increased from 0.615 for a single-domain to 0.705 for five-domain indices). This study further found that a minimalist 3-domain index performs as robustly as a 4- or 5-domain index. Another large multicentre European study involving 64,872 adults aged 50 and older from 15 countries further validated the structure and construct validity of IC, strengthening its generalisability beyond single-country cohorts.^24^ This study demonstrated that lower IC predicted subsequent declines in ADL and instrumental ADL over follow-up, independent of demographic and socioeconomic factors.

Interestingly, a study using data from the National Institute for Longevity Sciences–Longitudinal Study of Aging (NILS-LSA) in Japan and the Longitudinal Aging Study of Taipei (LAST) in Taiwan reported heterogenous findings regarding IC and mortality, highlighting potential sociocultural variation across populations.^25^ In this analysis of community-dwelling older adults aged ≥ 60 years (n = 794 in NILS-LSA and n = 1358 in LAST), IC impairment was assessed across locomotion, cognition, nutrition, sensory, and psychological domains. While IC impairment was associated with a higher risk of falls in both cohorts, the relationship with mortality differed: in the Japanese NILS-LSA cohort, men with impaired IC and visual impairment and women with poorer psychological well-being demonstrated increased mortality risk, whereas no statistically significant association between IC and mortality was observed in the Taiwanese LAST cohort.

The pattern we observed—where cognition, nutrition, and locomotor capacity most strongly predicted survival—is biologically plausible within the WHO IC framework, which conceptualises IC as the composite of mental and physical reserves that drive late-life functional trajectories. Lower cognitive reserve and faster cognitive decline have repeatedly been associated with increased mortality,^26^^,^^27^ probably through shared mechanisms involving vascular, metabolic, and neurodegenerative diseases, as well as downstream effects on self-management and adherence. Evidence from a large U.S. national cohort demonstrated that poorer cognitive performance predicted higher mortality even at working ages, with the relative risk strongest in midlife and persisting after adjustment for demographic, socioeconomic, and health factors, suggesting cognition may serve as an early marker of vulnerability long before dementia becomes clinically apparent.^26^ Similarly, long-term follow-up data from the UK Health and Lifestyle Survey showed that slower reaction times and poorer cognitive performance were associated with greater risk of cause-specific mortality, particularly from cardiovascular, stroke, and respiratory diseases, with stronger associations observed among individuals aged over 60 years.^27^

These associations likely reflect shared biological aging pathways rather than independent domain-specific effects. Cognitive decline, poor nutrition, and reduced locomotor capacity frequently co-occur due to common neurovascular, metabolic, and inflammatory mechanisms that affect both brain and peripheral systems. Cerebrovascular disease and neurodegenerative processes can simultaneously impair executive function and motor control, while chronic cardiometabolic conditions and low-grade systemic inflammation contribute to muscle catabolism, anorexia of aging, and reduced physiological reserve. In addition, cognitive impairment may indirectly increase mortality risk through behavioral pathways, including poor adherence to medical therapy, impaired self-care, and reduced ability to respond to acute illness. Together, these interrelated deficits may represent convergent manifestations of declining intrinsic capacity, capturing a state of heightened vulnerability that predisposes older adults to adverse outcomes and reduced survival.

Similarly, a slower gait speed—a concise marker of multisystem integrity including neuromuscular, cardiovascular, and central processing—has long been a strong predictor of survival.^28^^,^^29^ In the Rotterdam Study, detailed quantitative gait analysis demonstrated that multiple gait domains, particularly pace (reflecting gait speed), rhythm, and stance phases, were independently associated with higher all-cause and cause-specific mortality. Notably, each 0.1 m/s decrement in gait speed was associated with a 14–21% higher risk of death even after extensive adjustment for lifestyle factors, comorbidities, and neurological and cardiopulmonary health, and the association persisted among individuals free of major comorbidities, underscoring gait speed as a global marker of underlying physiological aging rather than disease burden alone.^28^ Complementing these findings, a large pooled analysis of over 34,000 community-dwelling older adults showed a graded, dose–response relationship between gait speed and long-term survival across the full spectrum of walking speeds, with predicted 10-year survival varying nearly four-fold across gait speed categories. Importantly, survival estimates incorporating gait speed were as accurate as those derived from models including multiple chronic conditions and functional measures, highlighting gait speed as a simple yet powerful integrative indicator of longevity.^29^

Nutritional status affects immunity, sarcopenia, and resilience to stressors, providing a mechanistic link to mortality risk.^30^^,^^31^ Evidence from very old adults with multimorbidity has shown that malnutrition assessed using established indices such as the Mini Nutritional Assessment (MNA), Geriatric Nutritional Risk Index, and Prognostic Nutritional Index independently predicted mortality, with hazard ratios ranging from approximately 1.6 to nearly 3, and significantly improved prognostic model performance, underscoring nutrition as a robust indicator of physiological reserve.^30^ Similarly, long-term follow-up of hospitalized older adults demonstrated that MNA-defined malnutrition and even risk of malnutrition were associated with persistently higher mortality across a 10-year period, with the greatest excess risk observed among those classified as malnourished at baseline, highlighting the sustained prognostic relevance of nutritional deficits beyond the acute care setting.^31^ Poor nutrition, reflecting both undernutrition and sarcopenia, contributes to immune dysregulation, chronic inflammation, reduced muscle mass, and impaired metabolic reserve, thereby limiting the ability to withstand acute illness and accelerating functional decline. Within the intrinsic capacity framework, nutritional impairment may therefore represent a central pathway linking biological vulnerability to adverse outcomes and reduced survival.

Impaired locomotor function diminishes mobility and independence, increasing the risk of accidents, hospitalisation, and accelerated functional decline. Cognitive impairment further exacerbates these risks by limiting an individual’s ability to plan meals, follow nutritional advice, stay physically active, and navigate their environment safely. Importantly, these domains are interconnected; There is growing recognition of biologically plausible mechanisms underlying this bidirectional relationship—such as the muscle–brain axis, where physical activity induces the release of myokines (e.g., brain-derived neurotrophic factor, irisin) that support neurogenesis, synaptic plasticity, and cognitive health. Conversely, neuroendocrine and inflammatory changes associated with cognitive decline can negatively impact muscle mass and function. Undernutrition can lead to sarcopenia and slower gait, while poor mobility restricts access to food and social engagement, worsening nutritional status. Similarly, both malnutrition and low physical activity are associated with faster cognitive decline through vascular, metabolic, and inflammatory pathways. This two-way relationship suggests that declines in cognition, nutrition, and mobility may interact to reduce intrinsic capacity, creating a self-perpetuating cycle that accelerates frailty and raises mortality risk.^32^^,^^33^

Interestingly, vision impairment was not associated with mortality after full adjustment (HR 1.08), a finding that aligns with mixed evidence across different countries. A large systematic review and meta-analysis including over 440,000 participants from 12 countries reported that vision impairment was associated with higher all-cause mortality, with pooled hazard ratios ranging from 1.29 for mild impairment (<6/12) to 1.89 for severe impairment (<6/60), suggesting a dose–response relationship with increasing severity.^34^ Moreover, substantial heterogeneity existed in vision assessment methods and analytical adjustments, with stronger associations observed in studies using best-corrected visual acuity, indicating that residual confounding and measurement differences may influence observed mortality risks. Together, these findings support the interpretation that vision impairment may act more as a marker of overall health and functional decline rather than an independent driver of mortality once broader intrinsic capacity and disease burden are fully accounted for. In our study, the lack of association may also relate to the method used to quantify vision, as visual function was operationalised as a continuous composite score derived from near and distance acuity in both eyes using logMAR-based measurements that were standardised into a z-score. While this approach captures subtle gradations in visual performance and aligns with the intrinsic capacity framework, it may attenuate threshold effects seen with categorical definitions of visual impairment used in prior studies, thereby potentially reducing observed mortality associations.

In contrast, hearing impairment remained modestly associated with mortality (HR 1.19), supporting evidence that sensory deprivation can increase the risk of death through pathways such as social isolation, cognitive decline, and reduced physical activity.^35^^,^^36^ Large U.S. cohort studies using audiometric assessments have shown that even mild hearing impairment is associated with a 20–40% higher risk of mortality after adjustment for demographic and cardiovascular factors, with a graded increase in risk at greater levels of hearing loss, reinforcing hearing impairment as an independent and clinically relevant marker of vulnerability in older adults.

Categorical analysis demonstrated a clear dose–response relationship between the number of impaired domains and mortality. Participants with one impaired domain faced a 1.5-fold higher risk of death (HR 1.48), those with two domains had a 2.1-fold increased risk (HR 2.10), and with four impaired domains, the risk exceeded threefold (HR 3.15), emphasising the cumulative effect of multidomain decline. These findings are consistent with previous studies, where multidomain IC deficits predicted mortality in a graded manner.^37^^,^^38^ Our study shows that these principles also hold for older adults in India, who encounter unique social, nutritional, and health challenges.

Most studies on IC from India are cross-sectional in design,^12^^,^^39^ whereas a study by Waris et al. examined the association between IC and functional outcomes such as ADL and IADL.^11^ Evidence on cognition and dementia is limited; a study based on the Global Burden of Diseases, Injuries, and Risk Factors Study 2021 cause-of-death data reported that dementia mortality increased steadily over time, rising with age and later birth cohorts, and was increasingly driven by metabolic risk factors such as high fasting plasma glucose and high BMI, while tobacco-attributable risk declined.^40^ Another study using the 10/66 Dementia Research Group cohort found that physical inactivity, dementia, depression, poor self-related health and disability were associated with higher mortality.^41^

While no study among community-dwelling Indian older adults has explored the association between nutrition, locomotion and mortality, a study among older Indian patients with cancer reported shorter overall survival in those with impaired timed-up-and-go performance.^42^ Similarly, evidence from a large geriatric oncology cohort in India demonstrated that impairments in nutrition, functional status, and cognition were independently associated with higher mortality.^33^

Future studies could explore whether intrinsic capacity predicts mortality more robustly than the number of comorbidities, further supporting a shift from a disease-centered to a function-centered approach in assessing older adults.

These findings highlight the potential public health benefit of regularly assessing IC in community settings as a practical, multidimensional measure of functional health and survival risk among older adults. In low- and middle-income countries like India—where the ageing population is rapidly increasing and health systems face resource limitations—IC-based screening could allow for the early detection of vulnerable individuals before visible disability appears. By emphasising specific domains such as cognition, nutrition, and locomotion as important predictors of mortality, our study offers clear targets for preventive measures and health promotion efforts. Incorporating IC assessment into primary care and community outreach programmes could help with risk stratification, guide personalised care plans, and support scalable, cost-effective models of healthy ageing that align with the WHO’s Decade of Healthy Ageing objectives.

This study utilises a large, cohort of older Indian adults with longitudinal follow-up, improving external validity for community-dwelling populations. Intrinsic capacity (IC) was evaluated using standardised, domain-specific instruments (comprehensive cognitive tests, CES-D, full MNA, 4-m gait speed, visual acuity, and a hearing screener), enhancing measurement accuracy across functional domains. We operationalised IC in multiple ways (continuous summated score and domain-wise impairment counts) and observed a clear dose–response relationship with mortality, which supports causal inference. Robust multivariable Cox models—alongside a model including all domains simultaneously—helped identify independent domain contributions beyond age, sex, BMI, and major chronic conditions. Mortality data collection through end-of-life interviews minimised loss of vital status information, and predefined rules for time-to-event analysis ensured consistent survival estimates. Although the conceptual framework linking intrinsic capacity to survival is well established, a key strength of this studies lies in its provision of longitudinal representative evidence from India, thereby extending the external validity of the intrinsic capacity paradigm to a large, underrepresented low-and middle-income country setting.

Comorbidities were self-reported (i.e., “known to the participant”) rather than objectively measured or clinically verified, which may lead to misclassification and residual confounding. Mortality dates were reported with month and year precision and standardised (using the first day of the month and mid-year or mid-interval assumptions when partially missing), potentially causing non-differential errors in survival times. IC and covariates were measured only at baseline; we did not account for time-varying changes or trajectories, so reverse causation (preterminal decline) cannot be entirely ruled out. Some domain measures may have limited scope: the hearing screener might miss mild loss compared with audiometry; the vision composite (summing near and distance acuity) does not include contrast sensitivity or visual fields. Additionally, data on cause of death, illness episodes, or hospitalisations were not collected, limiting further contextualisation of outcomes.

Although we adjusted for multiple sociodemographic and medical factors, unmeasured confounding (e.g., medication use, physical activity, diet quality, substance use, inflammation, or socioeconomic shocks) remains possible. Not all participants contributed data for every IC domain; if missingness disproportionately involved frailer or healthier individuals, estimates may be biased, and domain-specific analyses may have reduced precision. Selection into the LASI-DAD sub-sample and differential follow-up could introduce selection or attrition bias, potentially limiting generalisability to institutionalised or medically complex individuals not captured at baseline. Finally, the choice of a −1.5 SD cut-off to define domain impairment, while consistent with prior work, is partly arbitrary; nevertheless, our parallel analyses using continuous z-scores minimise dependence on any single threshold.

In conclusion, higher intrinsic capacity—particularly in cognition, nutrition, and locomotor function—is strongly associated with lower all-cause mortality among older Indian adults. The cumulative burden of impaired domains further increases risk, highlighting the importance of multidomain assessment. These findings, aligned with international studies including ELSA and HRS, support IC as a meaningful, actionable framework for promoting healthy ageing in India. By identifying specific domains associated with mortality risk, our study offers valuable evidence to inform public health policies and programmes aimed at maintaining intrinsic capacity and preventing premature death among older adults. Targeted interventions focusing on cognitive, nutritional, and physical domains may help reduce mortality and enhance quality of life in this ageing population.

## Contributors

ARR did the statistical analysis, wrote the first draft of the manuscript, MB did the interpretation of data analysis, wrote the first draft of the manuscript, ARR, MB, AR, MW, SK, SG, MV, SB, RM, TSR edited the manuscript, AC, JL, SD, and ABD edited the manuscript. All authors had full access to all the data in the study. At least two authors have accessed and verified the data (ARR and MB). All authors have approved the manuscript.

## Data sharing statement

The LASI-DAD data can be accessed for free at the Gateway to Global Aging website (g2gaging.org).

## Declaration of interests

We declare no competing interests.
