# Supplementary material for: Intrinsic capacity and survival among older adults in India: LASI-DAD study (wave 1 and wave 2)
Source: Lancet Reg Health Southeast Asia. 2026 May 9;49:100775. doi: 10.1016/j.lansea.2026.100775 (PMC13188132; doi:10.1016/j.lansea.2026.100775)
Supplement: Supplementary Figs. S1–S6 and Tables S1–S15 [file mmc1.pdf]

Intrinsic Capacity and Survival in Older Adults: Evidence from a Nationally Representative Cohort in India

Supplementary Materials

Supplementary Table 1: Measurement of the Cognition Domain

Supplementary Table 2: Measurement and coding of Intrinsic Capacity domains

Supplementary Table 3· Univariable survival analysis of demographic and comorbidities

Supplementary Table 4: Proportional Hazards Assumption assessed using Schoenfeld residual-based tests for IC and domains as continuous variables

Supplementary Table 5. Proportional Hazards Assumption assessed using Schoenfeld residual-based tests for IC and domains as categorical variables

Supplementary Table 6: Survival analysis of summated IC score and individual domains in Male

Supplementary Table 7: Survival analysis of summated IC score and individual domains in Female

Supplementary Table 8: Proportional Hazards Assumption assessed using Schoenfeld residual-based tests for IC and domains as continuous variables by sex

Supplementary Table 9: Survival analysis of intrinsic capacity and domains defined as categorical variable in Male

Supplementary Table 10: Survival analysis of intrinsic capacity and domains defined as categorical variable in Female

Supplementary Table 11. Proportional Hazards Assumption assessed using Schoenfeld residual-based tests for IC and domains as categorical variables by sex

Supplementary Table 12. Sensitivity analysis showing survival analysis of summated IC score and individual domain

Supplementary Table 13. Sensitivity analysis showing survival analysis of intrinsic capacity and domains defined as categorical variable

Supplementary Table 14: Proportional hazards assessed using Schoenfeld residual-based tests for sensitivity analyses using alternative censoring assumptions for IC and domains as continuous variables

Supplementary Table 15. Proportional hazards assessed using Schoenfeld residual-based tests for sensitivity analyses using alternative censoring assumptions for IC and domains as categorical variables

Supplementary Figure 1: Flowchart: Study Sample for Mortality Analysis (LASI-DAD)

Supplementary Figure 2: Deviance residuals plotted against the linear predictor for Cox proportional hazards models examining the association between intrinsic capacity and mortality. Panel A shows Model 3 with intrinsic capacity entered as a continuous variable, and Panel B shows Model 3 with intrinsic capacity entered as a categorical variable.

Supplementary Figure 3: Scaled Schoenfeld residual plots assessing the proportional hazards assumption for Model 3 with intrinsic capacity (IC) entered as a continuous variable.

Supplementary Figure 4: Scaled Schoenfeld residual plots assessing the proportional hazards assumption for Model 3 with individual intrinsic capacity domains entered as continuous variables: (A) Cognition, (B) Mood, (C) Nutrition, (D) Locomotion, (E) Vision, and (F) Hearing.

Supplementary Figure 5: Scaled Schoenfeld residual plots assessing the proportional hazards assumption for Model 3 with intrinsic capacity impairment entered as categorical variables according to the number of impaired domains: (A) One impaired domain, (B) Two impaired domains, (C) Three impaired domains, and (D) Four impaired domains.

Supplementary Figure 6: Scaled Schoenfeld residual plots assessing the proportional hazards assumption for Model 3 with individual intrinsic capacity domains entered as categorical variables: (A) Cognition, (B) Mood, (C) Nutrition, (D) Locomotion, (E) Vision, and (F) Hearing.

Supplementary Table 1: Measurement of the Cognition Domain

**Test / Assessment**	**Cognitive Ability Measured**	**Scoring / Transformation**
Hindi-Mental State Examination (HMSE)	Orientation, attention, immediate memory, language, visuospatial skills, executive function	Total score standardized (z-score)
Word List Recall (Immediate)	Verbal memory	Sum of correct words recalled across three trials; standardized (z-score)
Word List Recall (Delayed)	Long-term memory	Number of words recalled after a delay; standardized (z-score)
Word Recognition Test	Recognition memory	Number of correct identifications; standardized (z-score)
Story Recall (Immediate)	Episodic memory	Recall of key details immediately after story presentation; standardized (z-score)
Story Recall (Delayed)	Episodic memory	Recall of story details after a delay; standardized (z-score)
Verbal Fluency Test	Language, executive function	Number of correct animals named; standardized (z-score)
Object Naming / Identification (CSID)	Language, semantic memory	Correct identification of common objects; standardized (z-score)
Visual Recognition (RV Test)	Visuospatial memory	Number of correct answers identifying missing pieces in images; standardized (z-score)

Supplementary Table 2: Measurement and coding of Intrinsic Capacity domains

**Domain**	**Instrument / Item(s)**	**Measurement / Scoring**	**Transformation / Standardization**	**Final Score / z-score**	**Cut-off for Impairment**
Cognition	Standardized total cognition score derived from multiple tests covering: orientation, immediate & delayed word recall, story recall (immediate & delayed), verbal fluency, language comprehension, object naming, visuospatial ability, executive function	Raw scores from individual tests; total cognition score calculated by summing standardized components	Each test first standardized (z-score) using baseline population; summed to create total cognition score; higher = better cognition	z-score (higher = better cognition)	–1.5 SD below mean
Mood	Center for Epidemiologic Studies Depression Scale (CES-D)	Raw CES-D score (higher = worse mood)	Multiply z-score by –1 so higher values indicate better mood	z-score (higher = better mood)	–1.5 SD below mean
Nutrition	Mini Nutritional Assessment (MNA) full form	Total MNA score	Converted to z-score	z-score (higher = better nutrition)	–1.5 SD below mean
Locomotor	4-meter gait speed	Average of two trials in m/s	Converted to z-score	z-score (higher = better locomotor function)	–1.5 SD below mean
Vision	Near and distance visual acuity in right and left eyes (logMAR charts)	logMAR values for each eye and distance	Multiply by –1 so higher = better; sum 4 adjusted values; standardize	z-score (higher = better vision)	–1.5 SD below mean
Hearing	HearCheck Screener, 6 tones per ear	One point per tone detected; sum both ears	Standardize sum of tones as z-score	z-score (higher = better hearing)	–1.5 SD below mean

Supplementary Table 3· Univariable survival analysis of demographic and comorbidities

Variable	Hazard ratio	95% confidence interval	p-value
Age	1·074	1·067-1·082	<0·001
Sex			
Male	1 (ref)		
Female	0·669	0·589-0·760	<0·001
BMI	0·938	0·924-0·952	<0·001
Comorbidities			
Hypertension	1·142	1·003-1·300	0·044
Diabetes	1·415	1·215-1·648	<0·001
Cancer	1·941	1·165-3·233	0·011
Chronic lung disease	1·562	1·286-1·898	<0·001
Chronic heart disease	1·243	0·986-1·567	0·066
Stroke	2·314	1·748-3·061	<0·001
Neurological and psychiatric illness	1·639	1·218-2·204	0·001
High cholesterol	0·729	0·524-1·014	0·061
Functional status			
ADL	0·825	0·799-0·851	<0·001
IADL	0·816	0·795-0·838	<0·001

Supplementary Table 4: Proportional Hazards Assumption assessed using Schoenfeld residual-based tests for IC and domains as continuous variables

**Variable**	**Model 1***		**Model 2****		**Model 3*****	
	**χ^2**	**p-value**	**χ^2**	**p-value**	**χ^2**	**p-value**
Intrinsic capacity	0.18	0.670	4.42	0.219	14.61	0.263
Cognition	0.48	0.490	5.36	0.147	15.53	0.214
Mood	0.01	0.913	3.24	0.356	14.03	0.299
Nutrition	1.05	0.306	4.63	0.201	15.67	0.207
Locomotor	3.69	0.055	6.25	0.099	16.80	0.157
Vision	0.42	0.516	4.24	0.237	16.11	0.186
Hearing	0.57	0.451	4.36	0.225	14.74	0.256

*Unadjusted
**adjusted for age and sex
***adjusted for age, sex, BMI, hypertension, diabetes, cancer, chronic lung disease, chronic heart disease, stroke, neurological and psychiatric illness, high cholesterol

Supplementary Table 5. Proportional Hazards Assumption assessed using Schoenfeld residual-based tests for IC and domains as categorical variables

**Variable**	**Model 1***		**Model 2****		**Model 3*****	
	**χ^2**	**p-value**	**χ^2**	**p-value**	**χ^2**	**p-value**
Intrinsic capacity	4.41	0.492	9.29	0.232	18.39	0.302
Individual domains						
Cognition	0.62	0.433	5.83	0.120	17.36	0.137
Mood	0.01	0.937	3.21	0.359	14.49	0.271
Nutrition	0.48	0.488	3.88	0.275	15.34	0.223
Locomotor	1.61	0.205	3.75	0.289	14.61	0.263
Vision	0.29	0.590	3.93	0.269	15.71	0.205
Hearing	3.05	0.081	6.90	0.075	17.49	0.132

*Unadjusted
**adjusted for age and sex
***adjusted for age, sex, BMI, hypertension, diabetes, cancer, chronic lung disease, chronic heart disease, stroke, neurological and psychiatric illness, high cholesterol

Supplementary Table 6: Survival analysis of summated IC score and individual domains in Male

Variable	Model 1*		Model 2**		Model 3***	
	HR (95% CI)	p-value	HR (95% CI)	p-value	HR (95% CI)	p-value
Intrinsic capacity	0.84 (0.83-0.87)	<0.001	0.88 (0.86-0.91)	<0.001	0.88 (0.86-0.92)	<0.001
Cognition	0.64 (0.59-0.69)	<0.001	0.72 (0.66-0.79)	<0.001	0.70 (0.64-0.78)	<0.001
Mood	0.81 (0.74-0.88)	<0.001	0.86 (0.78-0.93)	0.001	0.89 (0.81-0.98)	0.020
Nutrition	0.67 (0.61-0.73)	<0.001	0.72 (0.65-0.79)	<0.001	0.72 (0.64-0.80)	<0.001
Locomotor	0.61 (0.56-0.67)	<0.001	0.71 (0.64-0.78)	<0.001	0.72 (0.65-0.80)	<0.001
Vision	0.85 (0.78-0.93)	0.001	0.94 (0.86-1.03)	0.193	0.95 (0.86-1.04)	0.284
Hearing	0.76 (0.69-0.83)	<0.001	0.86 (0.79-0.94)	0.001	0.88 (0.80-0.97)	0.014

*Unadjusted
**adjusted for age
***adjusted for age, BMI, hypertension, diabetes, cancer, chronic lung disease, chronic heart disease, stroke, neurological and psychiatric illness, high cholesterol

Supplementary Table 7: Survival analysis of summated IC score and individual domains in Female

Variable	Model 1*		Model 2**		Model 3***	
	HR (95% CI)	p-value	HR (95% CI)	p-value	HR (95% CI)	p-value
Intrinsic capacity	0.81 (0.79-0.84)	<0.001	0.86 (0.84-0.89)	<0.001	0.87 (0.84-0.91)	<0.001
Cognition	0.53 (0.48-0.58)	<0.001	0.64 (0.58-0.71)	<0.001	0.69 (0.62-0.78)	<0.001
Mood	0.79 (0.73-0.87)	<0.001	0.84 (0.77-0.92)	<0.001	0.81 (0.73-0.89)	<0.001
Nutrition	0.64 (0.58-0.71)	<0.001	0.71 (0.64-0.79)	<0.001	0.76 (0.67-0.86)	<0.001
Locomotor	0.58 (0.52-0.65)	<0.001	0.74 (0.64-0.84)	<0.001	0.74 (0.64-0.84)	<0.001
Vision	0.83 (0.75-0.92)	<0.001	0.91 (0.82-1.00)	0.052	0.93 (0.83-1.03)	0.158
Hearing	0.61 (0.55-0.67)	<0.001	0.72 (0.65-0.79)	<0.001	0.78 (0.69-0.87)	<0.001

*Unadjusted
**adjusted for age
***adjusted for age, BMI, hypertension, diabetes, cancer, chronic lung disease, chronic heart disease, stroke, neurological and psychiatric illness, high cholesterol

Supplementary Table 8: Proportional Hazards Assumption assessed using Schoenfeld residual-based tests for IC and domains as continuous variables by sex

Variable	Model 1*		Model 2**		Model 3***	
	χ^2	p-value	χ^2	p-value	χ^2	p-value
Male						
Intrinsic capacity	0.85	0.266	4.25	0.119	10.24	0.509
Cognition	0.24	0.623	2.51	0.285	9.17	0.606
Mood	0.21	0.644	1.95	0.376	7.87	0.725
Nutrition	2.90	0.088	3.72	0.156	12.13	0.354
Locomotor	3.77	0.052	3.57	0.167	10.71	0.468
Vision	0.03	0.866	0.80	0.669	7.32	0.772
Hearing	0.68	0.409	2.65	0.265	7.87	0.725
Female						
Intrinsic capacity	0.08	0.773	0.54	0.763	15.47	0.162
Cognition	0.92	0.337	1.22	0.543	14.71	0.196
Mood	0.01	0.982	0.32	0.851	15.17	0.175
Nutrition	0.01	0.951	0.29	0.864	15.80	0.149
Locomotor	0.83	0.363	1.37	0.504	16.46	0.125
Vision	0.39	0.531	1.02	0.599	17.06	0.106
Hearing	0.17	0.683	0.40	0.820	14.3	0.217

*Unadjusted
**adjusted for age and sex

***adjusted for age, sex, BMI, hypertension, diabetes, cancer, chronic lung disease, chronic heart disease, stroke, neurological and psychiatric illness, high cholesterol

Supplementary Table 9: Survival analysis of intrinsic capacity and domains defined as categorical variable in Male

Variable	Model 1*		Model 2**		Model 3***	
	HR (95% CI)	p-value	HR (95% CI)	p-value	HR (95% CI)	p-value
Intrinsic capacity						
No impairment	1 (ref)		1 (ref)		1 (ref)	
One impaired	1.67 (1.36-2.05)	<0.001	1.45 (1.18-1.78)	<0.001	1.51 (1.21-1.89)	<0.001
Two impaired	3.29 (2.46-4.38)	<0.001	2.21 (1.64-2.98)	<0.001	2.68 (1.91-3.76)	<0.001
Three impaired	2.37 (1.22-4.60)	0.011	1.41 (0.72-2.76)	0.312	2.23 (1.14-4.36)	0.020
Four impaired	28.41 (10.50-76.79)	<0.001	8.41 (3.04-23.29)	<0.001	17.16 (5.69-51.75)	<0.001
Individual domains						
Cognition	2.95 (2.23-3.90)	<0.001	2.02 (1.51-2.71)	<0.001	2.75 (1.98-3.83)	<0.001
Mood	1.65 (1.16-2.33)	0.005	1.52 (1.08-2.16)	0.017	1.25 (0.83-1.89)	0.286
Nutrition	2.22 (1.66-2.96)	<0.001	1.89 (1.42-2.54)	<0.001	1.82 (1.35-2.46)	<0.001
Locomotor	2.47 (1.77-3.44)	<0.001	1.52 (1.08-2.14)	0.017	2.11 (1.49-2.99)	<0.001
Vision	1.62 (1.20-2.19)	0.002	1.23 (0.90-1.67)	0.193	1.49 (1.09-2.04)	0.012
Hearing	1.68 (1.26-2.24)	<0.001	1.34 (1.00-1.79)	0.049	1.47 (1.04-2.06)	0.028

*Unadjusted

**adjusted for age

***adjusted for age, BMI, hypertension, diabetes, cancer, chronic lung disease, chronic heart disease, stroke, neurological and psychiatric illness, high cholesterol

Supplementary Table 10: Survival analysis of intrinsic capacity and domains defined as categorical variable in Female

Variable	Model 1*		Model 2**		Model 3***	
	HR (95% CI)	p-value	HR (95% CI)	p-value	HR (95% CI)	p-value
Intrinsic capacity						
No impairment	1 (ref)		1 (ref)		1 (ref)	
One impaired	2.08 (1.66-2.60)	<0.001	1.70 (1.36-2.14)	<0.001	1.92 (1.51-2.45)	<0.001
Two impaired	4.17 (3.23-5.39)	<0.001	2.77 (2.11-3.63)	<0.001	3.18 (2.35-4.30)	<0.001
Three impaired	4.44 (2.80-7.05)	<0.001	2.02 (1.24-3.28)	0.005	3.86 (2.35-6.32)	<0.001
Four impaired	5.88 (2.18-15.85)	<0.001	2.74 (1.01-7.44)	0.047	4.39 (1.61-12.01)	0.004
Individual domains						
Cognition	4.19 (3.35-5.24)	<0.001	2.42 (1.87-3.09)	<0.001	3.18 (2.44-4.16)	<0.001
Mood	1.63 (1.23-2.15)	0.001	1.46 (1.11-1.93)	0.007	1.76 (1.30-2.37)	<0.001
Nutrition	2.44 (1.85-3.22)	<0.001	2.00 (1.51-2.65)	<0.001	1.83 (1.37-2.45)	<0.001
Locomotor	2.86 (2.14-3.82)	<0.001	1.44 (1.04-1.99)	0.030	2.62 (1.94-3.55)	<0.001
Vision	1.56 (1.13-2.14)	0.006	1.21 (0.88-1.67)	0.241	1.37 (0.98-1.92)	0.067
Hearing	2.57 (1.97-3.35)	<0.001	1.77 (1.35-2.33)	<0.001	1.75 (1.25-2.45)	0.001

*Unadjusted

**adjusted for age

***adjusted for age, BMI, hypertension, diabetes, cancer, chronic lung disease, chronic heart disease, stroke, neurological and psychiatric illness, high cholesterol

Supplementary Table 11. Proportional Hazards Assumption assessed using Schoenfeld residual-based tests for IC and domains as categorical variables by sex

**Variable**	**Model 1***		**Model 2****		**Model 3*****	
	**χ^2**	**p-value**	**χ^2**	**p-value**	**χ^2**	**p-value**
Male						
Intrinsic capacity	1.91	0.861	5.54	0.354	11.56	0.642
Individual domains						
Cognition	0.70	0.402	3.01	0.222	10.64	0.474
Mood	0.07	0.787	1.78	0.409	7.73	0.737
Nutrition	0.32	0.569	0.98	0.612	8.77	0.643
Locomotor	0.44	0.506	0.21	0.898	7.21	0.782
Vision	1.11	0.291	1.54	0.464	8.53	0.665
Hearing	5.83	0.016	7.54	0.023	11.58	0.396
Female						
Intrinsic capacity	3.03	0.696	2.75	0.839	16.52	0.348
Individual domains						
Cognition	0.52	0.469	1.01	0.603	14.38	0.212
Mood	0.02	0.900	0.24	0.886	15.28	0.169
Nutrition	0.36	0.547	0.29	0.863	13.32	0.273
Locomotor	1.69	0.194	2.21	0.331	16.13	0.136
Vision	0.11	0.739	0.50	0.778	15.65	0.154
Hearing	0.07	0.792	0.32	0.851	14.55	0.204

*Unadjusted
**adjusted for age and sex
***adjusted for age, sex, BMI, hypertension, diabetes, cancer, chronic lung disease, chronic heart disease, stroke, neurological and psychiatric illness, high cholesterol

Supplementary Table 12. Sensitivity analysis showing survival analysis of summated IC score and individual domain

**Variable**	**Model 1***		**Model 2****		**Model 3*****	
	**HR (95% CI)**	**p-value**	**HR (95% CI)**	**p-value**	**HR (95% CI)**	**p-value**
Intrinsic capacity	0.84 (0.83-0.86)	<0.001	0.87 (0.85-0.89)	<0.001	0.88 (0.86-0.90)	<0.001
Cognition	0.61 (0.57-0.65)	<0.001	0.68 (0.64-0.73)	<0.001	0.69 (0.64-0.75)	<0.001
Mood	0.84 (0.89-0.89)	<0.001	0.85 (0.79-0.90)	<0.001	0.86 (0.79-0.92)	<0.001
Nutrition	0.68 (0.63-0.73)	<0.001	0.72 (0.67-0.77)	<0.001	0.75 (0.69-0.81)	<0.001
Locomotor	0.64 (0.59-0.69)	<0.001	0.72 (0.66-0.78)	<0.001	0.73 (0.67-0.79)	<0.001
Vision	0.86 (0.81-0.92)	<0.001	0.93 (0.87-0.99)	0.038	0.94 (0.88-1.01)	0.104
Hearing	0.68 (0.64-0.72)	<0.001	0.78 (0.73-0.83)	<0.001	0.82 (0.76-0.88)	<0.001

*Unadjusted
**adjusted for age and sex
***adjusted for age, sex, BMI, hypertension, diabetes, cancer, chronic lung disease, chronic heart disease, stroke, neurological and psychiatric illness, high cholesterol

Supplementary Table 13. Sensitivity analysis showing survival analysis of intrinsic capacity and domains defined as categorical variable

**Variable**	**Median survival (months)**	**Model 1***		**Model 2****		**Model 3*****	
		**HR (95% CI)**	**p-value**	**HR (95% CI)**	**p-value**	**HR (95% CI)**	**p-value**
Intrinsic capacity							
No impairment	Not reached	1 (ref)		1 (ref)			
One impaired	Not reached	1·76 (1·52-2·04)	<0·001	1·56 (1·34-1·82)	<0·001	1·50 (1·27-1·77)	<0·001
Two impaired	50.03	3·42 (2·84-4·13)	<0·001	2·49 (2·04-3·04)	<0·001	2·14 (1·69-2·69)	<0·001
Three impaired	56.96	3·27 (2·25-4·76)	<0·001	1·91 (1·29-2·82)	0·001	1·87 (1·24-2·81)	0·003
Four impaired	22.01	9·52 (4·73-19·15)	<0·001	5·26 (2·59-10·68)	<0·001	4·21 (2·04-8·69)	<0·001
Individual domains	N						
Cognition	N=4095	3·47 (2·92-4·12)	<0·001	2·32 (1·92-2·79)	<0·001	2·19 (1·77-2·72)	<0·001
Mood	N=4094	1·53 (1·23-1·89)	<0·001	1·52 (1·23-1·89)	<0·001	1·53 (1·20-1·94)	0·001
Nutrition	N=3623	2·17 (1·78-2·65)	<0·001	1·91 (1·56-2·34)	<0·001	1·68 (1·36-2·07)	<0·001
Locomotor	N=3619	2·47 (1·99-3·07)	<0·001	1·47 (1·16-1·86)	0·001	1·33 (1·04-1·70)	0·023
Vision	N=3716	1·54 (1·24-1·92)	<0·001	1·22 (0·98-1·52)	0·076	1·16 (0·92-1·47)	0·198
Hearing	N=4096	2·19 (1·80-2·66)	<0·001	1·64 (1·35-2·00)	<0·001	1·39 (1·09-1·77)	0·007

*Unadjusted
**adjusted for age and sex
***adjusted for age, sex, BMI, hypertension, diabetes, cancer, chronic lung disease, chronic heart disease, stroke, neurological and psychiatric illness, high cholesterol

Supplementary Table 14: Proportional hazards assessed using Schoenfeld residual-based tests for sensitivity analyses using alternative censoring assumptions for IC and domains as continuous variables

Variable	Model 1*		Model 2**		Model 3***	
	χ^2	p-value	χ^2	p-value	χ^2	p-value
Intrinsic capacity	0.05	0.825	4.66	0.198	15.52	0.214
Cognition	0.27	0.601	5.56	0.135	16.01	0.191
Mood	0.01	0.905	3.57	0.312	14.92	0.246
Nutrition	1.40	0.237	5.08	0.166	16.73	0.159
Locomotor	3.60	0.058	6.38	0.095	17.24	0.141
Vision	0.42	0.518	4.71	0.194	16.89	0.154
Hearing	0.17	0.684	4.28	0.233	15.54	0.213

*Unadjusted
**adjusted for age and sex
***adjusted for age, sex, BMI, hypertension, diabetes, cancer, chronic lung disease, chronic heart disease, stroke, neurological and psychiatric illness, high cholesterol

Supplementary Table 15. Proportional hazards assessed using Schoenfeld residual-based tests for sensitivity analyses using alternative censoring assumptions for IC and domains as categorical variables

Variable	Model 1*		Model 2**		Model 3***	
	χ^2	p-value	χ^2	p-value	χ^2	p-value
Intrinsic capacity	2.16	0.827	7.51	0.378	17.55	0.351
Individual domains						
Cognition	0.24	0.624	5.73	0.125	17.37	0.136
Mood	0.02	0.886	3.62	0.305	15.71	0.205
Nutrition	0.61	0.435	4.05	0.256	16.31	0.177
Locomotor	1.65	0.199	4.00	0.261	15.26	0.227
Vision	0.30	0.581	4.40	0.222	16.49	0.170
Hearing	1.81	0.179	6.10	0.107	17.41	0.135

*Unadjusted
**adjusted for age and sex
***adjusted for age, sex, BMI, hypertension, diabetes, cancer, chronic lung disease, chronic heart disease, stroke, neurological and psychiatric illness, high cholesterol

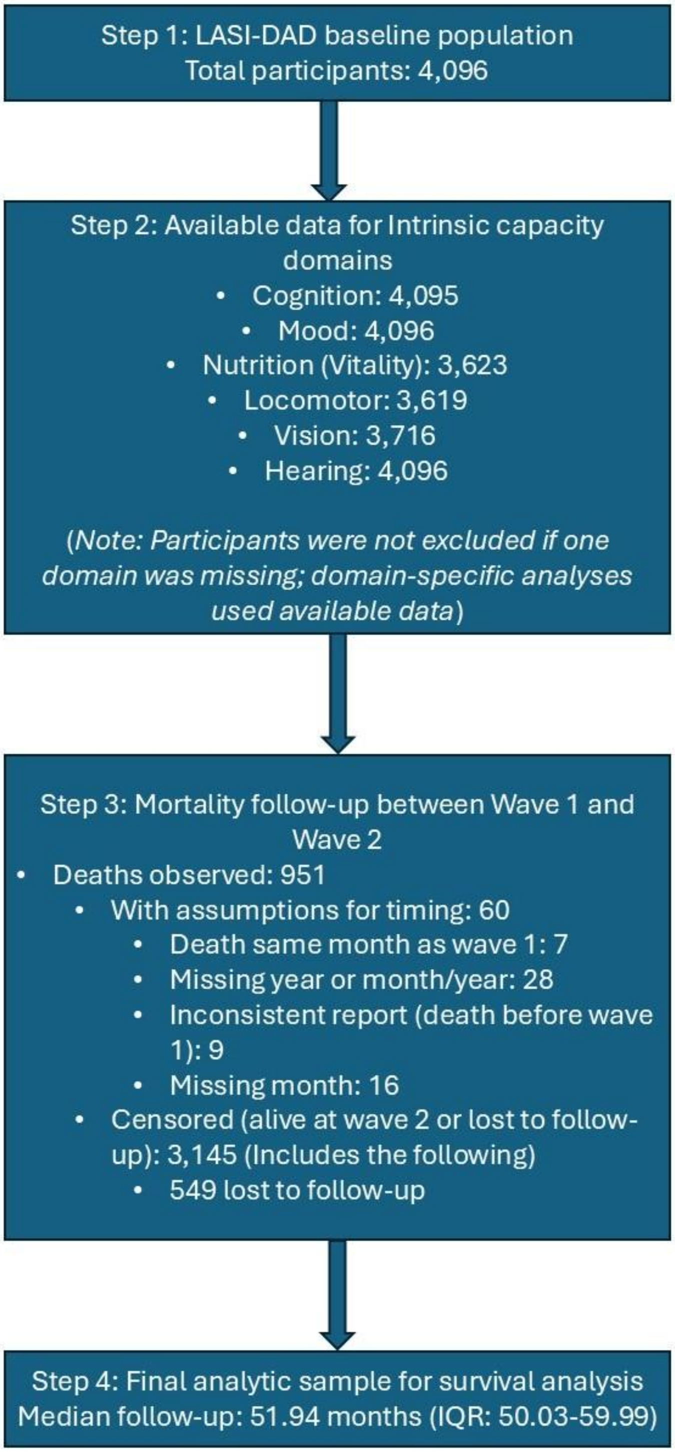

Supplementary Figure 1: Flowchart: Study Sample for Mortality Analysis (LASI-DAD)

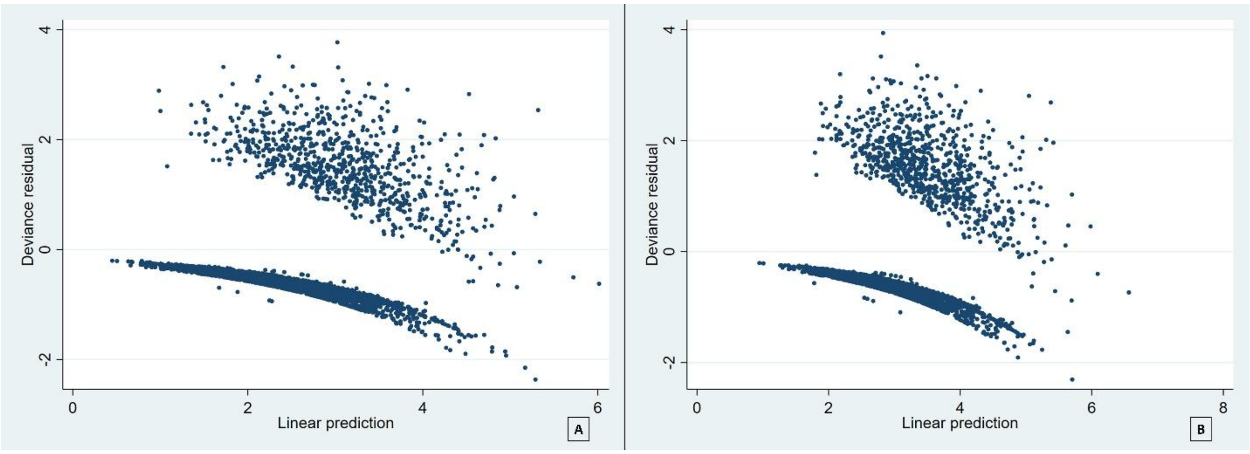

Supplementary Figure 2: Deviance residuals plotted against the linear predictor for Cox proportional hazards models examining the association between intrinsic capacity and mortality. Panel A shows Model 3 with intrinsic capacity entered as a continuous variable, and Panel B shows Model 3 with intrinsic capacity entered as a categorical variable.

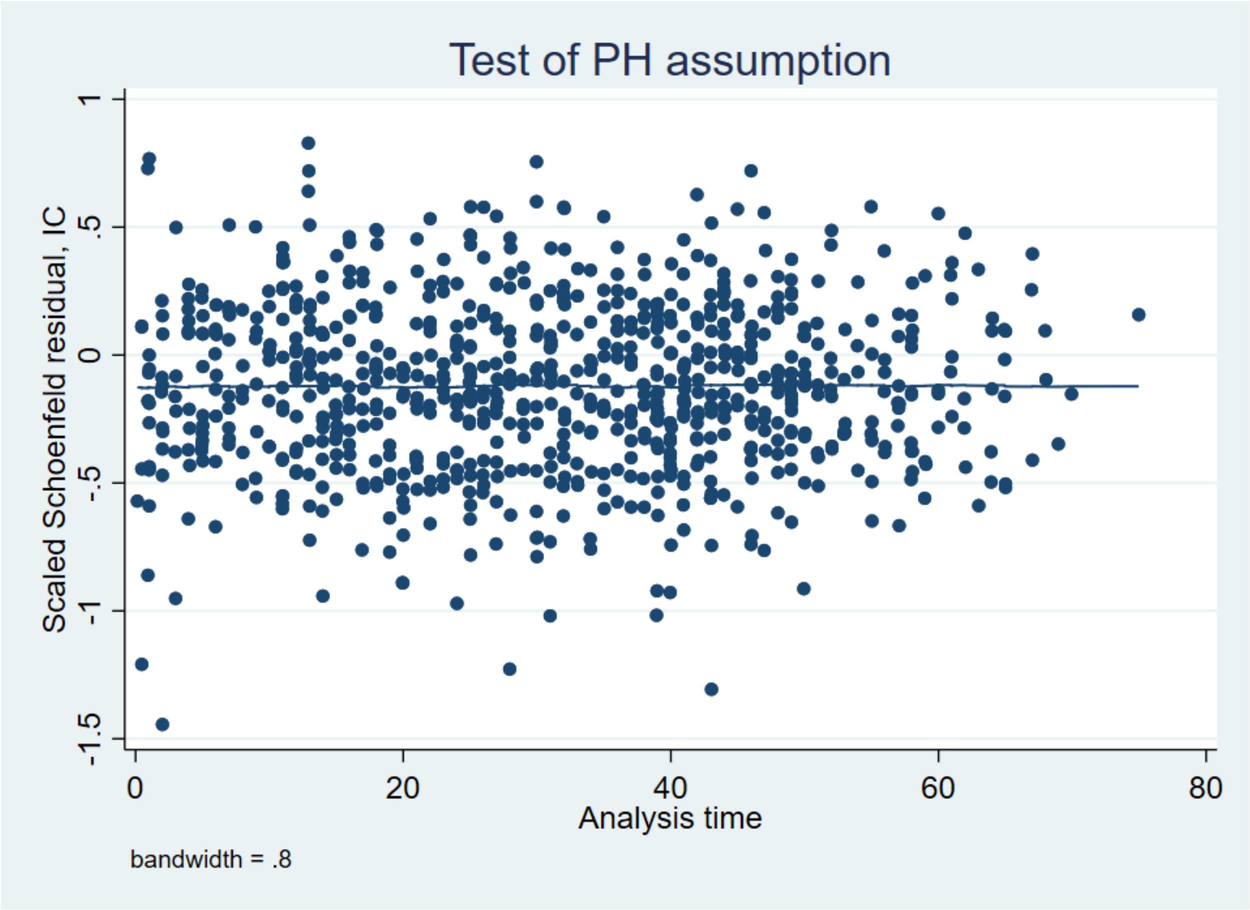

Supplementary Figure 3: Scaled Schoenfeld residual plots assessing the proportional hazards assumption for Model 3 with intrinsic capacity (IC) entered as a continuous variable.

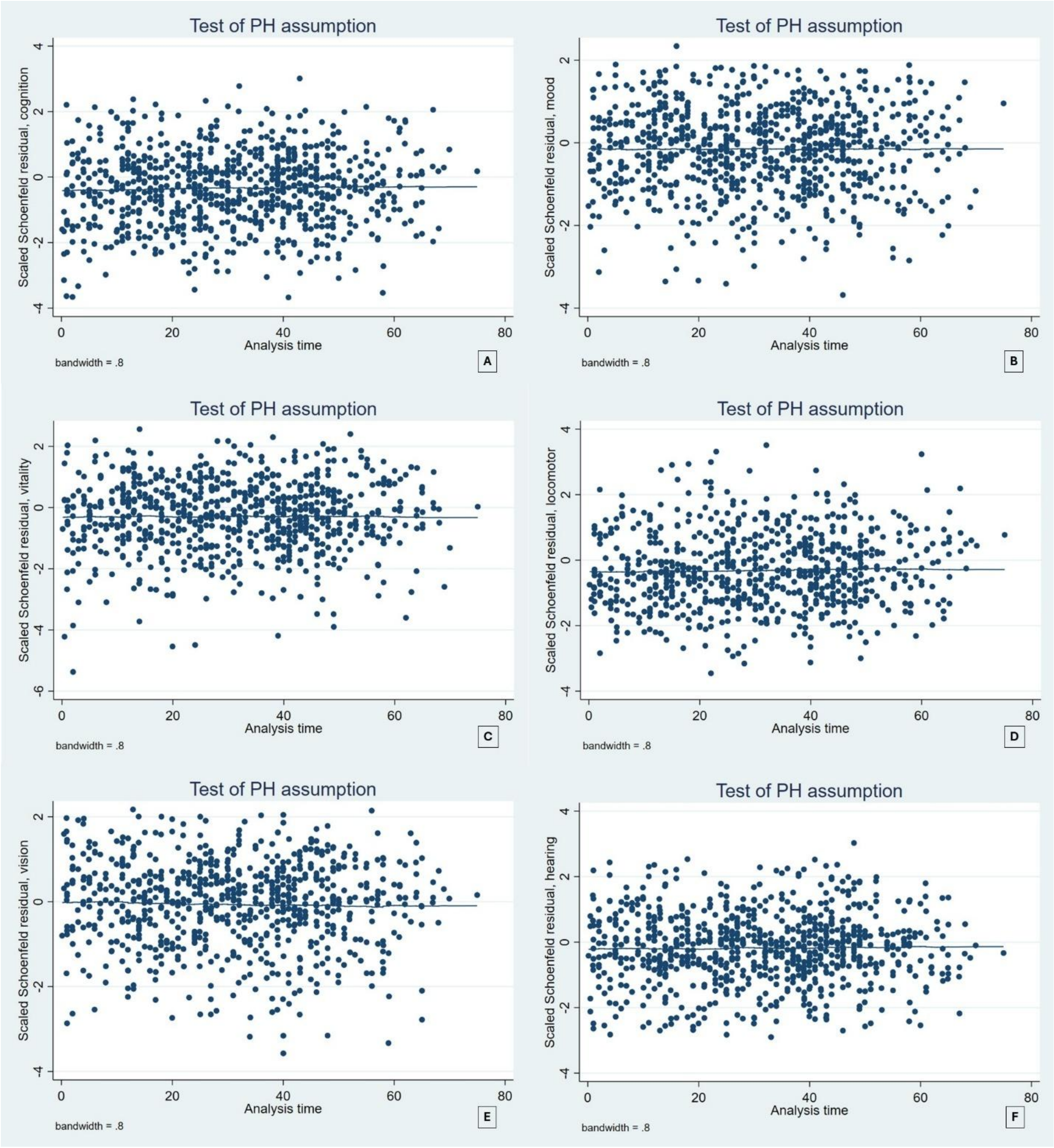

Supplementary Figure 4: Scaled Schoenfeld residual plots assessing the proportional hazards assumption for Model 3 with individual intrinsic capacity domains entered as continuous variables: (A) Cognition, (B) Mood, (C) Nutrition, (D) Locomotion, (E) Vision, and (F) Hearing.

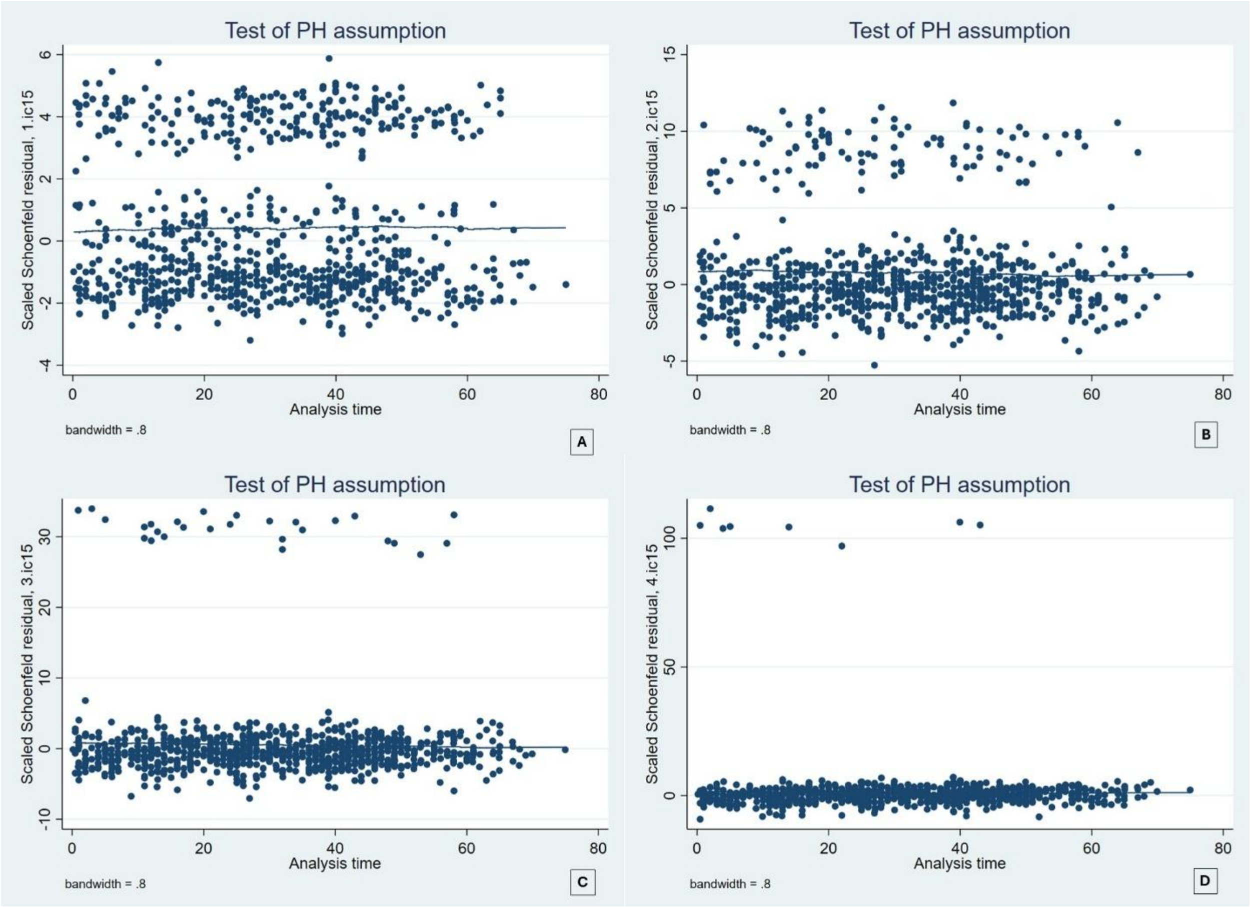

Supplementary Figure 5: Scaled Schoenfeld residual plots assessing the proportional hazards assumption for Model 3 with intrinsic capacity impairment entered as categorical variables according to the number of impaired domains: (A) One impaired domain, (B) Two impaired domains, (C) Three impaired domains, and (D) Four impaired domains.

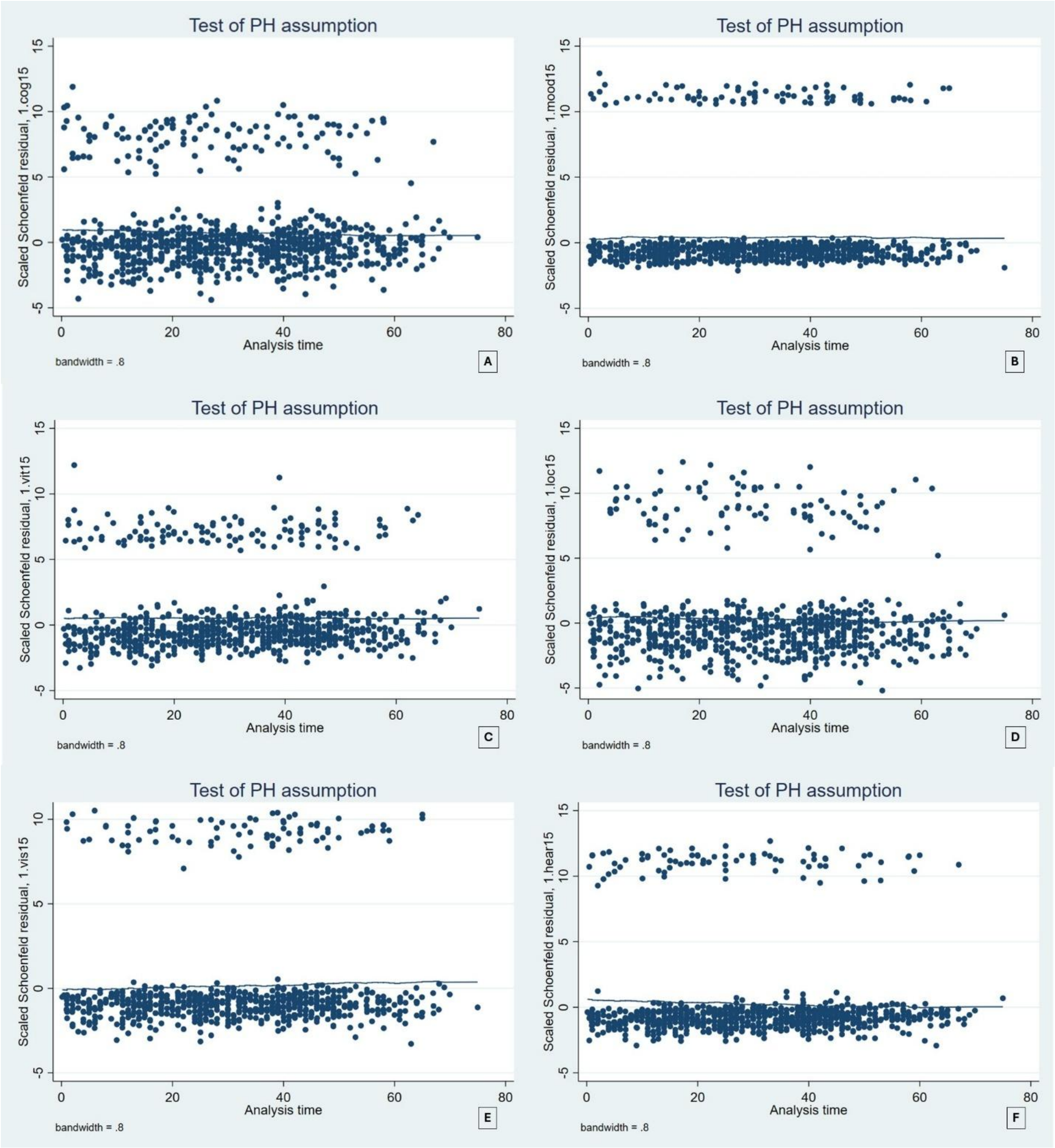

Supplementary Figure 6: Scaled Schoenfeld residual plots assessing the proportional hazards assumption for Model 3 with individual intrinsic capacity domains entered as categorical variables: (A) Cognition, (B) Mood, (C) Nutrition, (D) Locomotion, (E) Vision, and (F) Hearing.

:
